# In This Issue

**Published:** 1995

## GENETIC INFLUENCES ON ALCOHOLISM RISK: A REVIEW OF ADOPTION AND TWIN STUDIES

Adoption and twin studies have long been used to study the relative importance of genetic and environmental influences on the development of alcoholism. Dr. Andrew C. Heath recently completed a reanalysis of this large body of literature. His research confirms the existence of a significant genetic influence on alcoholism. Moreover, these genetic factors have remained remarkably consistent over time, whether the comparison is of U.S. male twins born in the 1920’s or U.S. adoptees born in the 1940’s, 1950’s, and 1960’s; or whether the study is of Swedish female adoptees born from 1930 to 1949 or Swedish female twins born as late as 1967. Dr. Heath summarizes the findings from his reanalysis of twin and adoption data and discusses the possible limitations of these studies. (pp. 166–171)

## A LONG-TERM STUDY OF SONS OF ALCOHOLICS

A classic approach to examining genetic factors in alcoholism has been to study alcoholic fathers and their sons. In this article, Dr. Marc A. Schuckit reviews data from a series of studies spanning 25 years. These long-term studies are proving especially useful for identifying specific alcohol-related traits that might interact with the environment to increase a person’s risk for developing alcohol problems. Dr. Schuckit describes the most recent results, comparing men with family histories of alcoholism and those with no such histories. A major finding from this research is that young men who had low responses to alcohol (i.e., who required more alcohol to experience an effect) had higher rates of alcohol problems later in life, regardless of their family histories. (pp. 172–175)

## THE SEARCH FOR BIOCHEMICAL MARKERS

Genetically determined biochemical markers of alcoholism could help researchers and treatment providers identify people who are predisposed to alcoholism. Such markers would improve prevention, diagnosis, and treatment efforts as well as facilitate research into the genetic basis of alcoholism. Two markers now under investigation are the activity levels of the enzymes monoamine oxygenase and adenylyl cyclase. These markers are inherited and frequently are lower in alcoholic than in nonalcoholic subjects. Although the activity levels of these enzymes cannot be used to distinguish the entire spectrum of alcoholics, Drs. Robert M. Anthenelli and Boris Tabakoff report that the markers may be useful in defining certain subtypes of alcoholics. (pp. 176–181)

## GENETIC INFLUENCES AFFECTING ALCOHOL USE AMONG ASIANS

The body processes alcohol using two important enzymes, alcohol dehydrogenase and aldehyde dehydrogenase, both of which are genetically controlled. Impaired function of these enzymes can strongly affect how a person feels when he or she consumes alcohol. For example, people with a defective aldehyde dehydrogenase gene respond to alcohol consumption with intense flushing and other physical symptoms, such as nausea. Because of the unpleasant side effects, these people generally consume less alcohol and are at a lower risk for developing alcoholism than people with functional genes. People of Asian heritage are more likely to have a defect in the aldehyde dehydrogenase gene than are those of other ethnic groups. Drs. Tamara L. Wall and Cindy L. Ehlers review studies of Asian subjects showing that people who inherit the defective gene from one or both parents have different physiological, psychological, and electrophysiological responses to alcohol than do people who have the functional gene. (pp. 184–189)

## THE HUMAN GENOME PROJECT

Nearly all human diseases, including alcoholism, can be traced, at least in part, to alterations in one or more genes. Thus, identifying and understanding all the genes that specify the body’s functions could provide scientists with powerful tools with which to understand, prevent, and treat diseases. An international research effort, the Human Genome Project, is now under way to decipher the chemical makeup of the entire human genetic code (i.e., the genome), to isolate all its genes, and to analyze the genes’ functions. Dr. Francis S. Collins and Leslie Fink summarize the project’s goals and recent progress. Although the project still is far from completion, researchers already have reached important milestones. The stage is now set for identifying the tens of thousands of remaining human genes and for using the newly gained knowledge in ways that are both responsible and ethical. (pp. 190–195)

## ADOPTION STUDIES

Since the beginning of this century, researchers have studied children who were separated from their biological families and raised in adoptive homes to determine the effects of genetic and environmental factors on certain characteristics, such as intelligence. According to Dr. Remi J. Cadoret, adoption studies are especially useful for assessing the relative contributions of genes, the environment, and gene-environment interactions in the development of alcoholism. Despite the significant impact that adoption studies have had on our understanding of the causes of alcohol dependence, Dr. Cadoret notes that confounding environmental factors, such as selective placement—in which children are preferentially placed in traditional, well-established, two-parent homes—can limit the usefulness of such studies if researchers fail to give such variables adequate consideration. (pp. 195–200)

## TWIN STUDY DESIGN

Twins offer a unique resource for evaluating the genetic aspects of behavior. Twins may be either identical, and thereby have the same genetic information, or fraternal, with only a portion of their genetic makeup in common. When a certain behavior or trait, such as alcoholism, is shared by both twins, it provides researchers with a unique opportunity for determining the degree to which that behavior is linked to a genetic influence. Drs. Carol A. Prescott and Kenneth S. Kendler describe how twin studies are conducted. They also review findings on the genetics of alcoholism from studies of male, female, and opposite-sex twin pairs. (pp. 200–205)

## GENETIC ENGINEERING IN ANIMAL MODELS

What role do individual genes play in the predisposition to alcohol dependence? Thanks to genetic engineering techniques now being pioneered in laboratory animals, researchers are making progress in identifying specific genes that contribute to the development of alcoholism in humans. Drs. Susanne Hiller-Sturmhöfel, Barbara J. Bowers, and Jeanne M. Wehner describe the potential usefulness as well as the limitations of techniques such as transgenic and knockout mice and antisense RNA strategies. Though the use of these technologies is still in its infancy in alcohol research, studies from other fields already have shed light on several complex physiological processes, such as the way alcohol affects the body’s response to stress. (pp. 206–213)

## PRIMATES IN ALCOHOL RESEARCH

Humans and primates share a large percentage of their genetic material and often display comparable complex social behaviors. Because of these similarities, primates frequently have been used to study human psychiatric syndromes. Only recently, however, have these animals appeared as subjects in alcohol research. In this article, Dr. J. Dee Higley reviews the use of nonhuman primates in alcohol research. He describes how this animal model of human behavior is providing insight into the genetic and environmental components that influence alcohol consumption and dependence. (pp. 213–216)

## MOLECULAR BIOLOGY

Although family, twin, and adoption studies have established a genetic contribution to alcoholism, the nature of this association is unknown. To better identify the role of genetics in people who are predisposed to alcoholism, researchers are turning to molecular biology techniques. In this article, Dr. Alison M. Goate describes the use of two such techniques. Positional cloning allows researchers to identify genes linked to specific diseases based solely on their location within the subject’s genetic material (i.e., genome). Actual genes implicated in the disease process (i.e., candidate genes) are then selected for analysis based on the positional cloning data. As the Human Genome Project continues to chart unknown sections of the genetic map, more disease-linked genes will be identified using a combination of positional cloning and the candidate gene approach. (pp. 217–220)

## QUANTITATIVE TRAIT LOCI MAPPING

Alcoholism is thought to be influenced by many genes located throughout the human genetic material (i.e., the genome). These genes are linked to certain characteristics, or traits, that are believed to influence the body’s responses to alcohol. Such traits are called quantitative because they are influenced by several genes; each gene, however, affects the overall characteristic to only a certain extent. Quantitative trait loci (QTL) analysis provides a means of locating and measuring the effects of a single QTL on a behavioral trait, such as alcoholism. In this article, Drs. Judith E. Grisel and John C. Crabbe provide a brief overview of the methods involved in QTL analyses and include several examples demonstrating the application of the technique. (pp. 220–227)

## THE COLLABORATIVE STUDY ON THE GENETICS OF ALCOHOLISM

In 1989 the National Institute on Alcohol Abuse and Alcoholism initiated the Collaborative Study on the Genetics of Alcoholism (COGA), a multidisciplinary, multicenter study to identify and analyze genetic factors contributing to a person’s risk for alcoholism. In this special section, senior COGA investigators highlight preliminary findings gleaned from the ambitious project. The authors describe the history and design of COGA, emphasize the importance of accurate clinical assessment of alcoholism, summarize data from neurophysiological and alcohol administration experiments, review research progress regarding biochemical and molecular genetic markers, and provide an overview of the data analysis approaches being used. (pp. 228–236)

## ALCOHOL’S EFFECTS ON GENE EXPRESSION

Not only does a person’s genetic makeup help determine his or her response to alcohol consumption, alcohol, in turn, can alter the expression of certain genes. Scientists have developed several methods to isolate and identify such alcohol-regulated genes. Dr. Michael F. Miles examines a few of these techniques and the genes scientists have helped to identify. These genes, which are involved in a variety of physiological functions, such as cellular communication, could play a pivotal role in the brain’s adaptation to alcohol. Further study will enable scientists to better understand the mechanisms through which alcohol might modify the expression of these genes. (pp. 237–243)

